# Contrasting effects of comorbidities on emergency colon cancer diagnosis: a longitudinal data-linkage study in England

**DOI:** 10.1186/s12913-019-4075-4

**Published:** 2019-05-15

**Authors:** Cristina Renzi, Georgios Lyratzopoulos, Willie Hamilton, Camille Maringe, Bernard Rachet

**Affiliations:** 1 Department of Behavioural Science and Health, ECHO (Epidemiology of Cancer Healthcare & Outcomes) Research Group, University College London, 1-19 Torrington Place, London, WC1E 6BT UK; 2 Department of Non-communicable Disease Epidemiology, Cancer Survival Group, London School of Hygiene and Tropical Medicine, Keppel St, Bloomsbury, London, WC1E 7HT UK; 3 University of Exeter Medical School, St Luke’s Campus, Heavitree Road, Exeter, EX1 2LU UK

**Keywords:** Colon cancer, Comorbidity, Chronic conditions, Emergency presentations, Diagnosis, Symptoms

## Abstract

**Background:**

One in three colon cancers are diagnosed as an emergency, which is associated with worse cancer outcomes. Chronic conditions (comorbidities) affect large proportions of adults and they might influence the risk of emergency presentations (EP).

**Methods:**

We aimed to evaluate the effect of specific pre-existing comorbidities on the risk of colon cancer being diagnosed following an EP rather than through non-emergency routes. The cohort study included 5745 colon cancer patients diagnosed in England 2005–2010, with individually-linked cancer registry, primary and secondary care data. In addition to multivariable analyses we also used potential-outcomes methods.

**Results:**

Colon cancer patients with comorbidities consulted their GP more frequently with cancer symptoms during the pre-diagnostic year, compared with non-comorbid cancer patients. EP occurred more frequently in patients with ‘serious’ or complex comorbidities (diabetes, cardiac and respiratory diseases) diagnosed/treated in hospital during the years pre-cancer diagnosis (43% EP in comorbid versus 27% in non-comorbid individuals; multivariable analysis Odds Ratio (OR), controlling for socio-demographic factors and symptoms: men OR = 2.40; 95% CI 2.0–2.9 and women OR = 1.98; 95% CI 1.6–2.4. Among women younger than 60, gynaecological (OR = 3.41; 95% CI 1.2–9.9) or recent onset gastro-intestinal conditions (OR = 2.84; 95% CI 1.1–7.7) increased the risk of EP. In contrast, primary care visits for hypertension monitoring decreased EPs for both genders.

**Conclusions:**

Patients with comorbidities have a greater risk of being diagnosed with cancer as an emergency, although they consult more frequently with cancer symptoms during the year pre-cancer diagnosis. This suggests that comorbidities may interfere with diagnostic reasoning or investigations due to ‘competing demands’ or because they provide ‘alternative explanations’. In contrast, the management of chronic risk factors such as hypertension may offer opportunities for earlier diagnosis. Interventions are needed to support the diagnostic process in comorbid patients. Appropriate guidelines and diagnostic services to support the evaluation of new or changing symptoms in comorbid patients may be useful.

**Electronic supplementary material:**

The online version of this article (10.1186/s12913-019-4075-4) contains supplementary material, which is available to authorized users.

## Background

According to international data, emergency diagnoses occur in up to 33% of colorectal cancers [1], with a higher risk for colon (31%) than rectal cancers (15%) [2]. Reducing emergency presentations is an important public health target [3], as they are associated with poor cancer survival, independently of stage at diagnosis [1], worse patient experience [4] and disruptions to hospital services [5]. One-year survival is 49% after emergency diagnosis of colorectal cancer compared to more than 80% for non-emergency routes [6]. Although some emergency diagnoses may be unavoidable, for example in rapidly progressing cancers with minimal or no prodromal symptoms until an acute presentation [1, 7], in a large proportion of cases emergency presenters have consulted their doctor with relevant symptoms during the months before the emergency cancer diagnosis [8–12]. Socio-economically deprived individuals, women, the youngest and oldest age-groups have increased risk of emergency presentations [1, 2, 8, 13], but population-based evidence on why such inequalities occur and how to reduce them is scant. Less frequent help-seeking for cancer symptoms and diagnostic delays due to atypical presentations are possible contributing factors.

Chronic conditions (hereafter called comorbidities) affect more than 50% of older adults [14–16]. Comorbidities might confer a higher risk of emergency cancer diagnosis [1], but current evidence is limited and mostly relates to studies evaluating the overall presence and number of comorbidities, without consideration of morbidity type and potential effect modification by presenting symptom and socio-demographic characteristics. Comorbidities providing ‘alternative’ explanations and those interfering with the cancer diagnosis through ‘competing demands’ (being unrelated to cancer but competing for clinical attention) can be associated with longer diagnostic intervals [17], but their specific effects on emergency presentations is unknown. Some comorbidities requiring regular follow-up visits might offer opportunities for earlier diagnosis [18].

In this study, using linked cancer registry, primary and secondary care data with clinical information for up to 10 years pre-cancer [8], we aimed to provide population-based evidence on the effect of specific comorbidities on primary care consultations for cancer-related symptoms during the year before a colon cancer diagnosis. We also aimed to estimate the effect of specific comorbidities on the risk of cancer being diagnosed through emergency rather than non-emergency routes, accounting for socio-demographic factors and symptoms.

When using observational data for estimating average effects in the population, traditional epidemiological methods can lead to biased results due to non-comparability of examined groups. Potential-outcomes or counterfactual approaches can overcome this limitation. Such approaches allowing to emulate randomized studies using observational data [19–21] are increasingly used for estimating treatment effects. They are also valuable for primary care and public health research [22]. When examining complex factors, for which many possible interventions exist, it is challenging to estimate causal effects [23] and in such circumstances potential-outcomes are particularly useful to clarify the relevance of the issue under examination [24–26] and critically consider the complex relationships between exposures and outcomes.

Thus, in addition to using conventional methods, we aimed to employ potential-outcomes approaches for determining the effects of specific comorbidities on the risk of cancer being diagnosed through emergency rather than non-emergency routes.

## Methods

### Study population and data sources

We included colon cancers (ICD-10 C18, further classified into distal C18.5-C18.7 and proximal C18.0-C18.4 [27–29] tumours) diagnosed in England 2005–2010 recorded in the Cancer Registry and linked to primary care (Clinical Practice Research Datalink-CPRD) and secondary care data (Hospital Episode Statistics-HES). CPRD provides prospectively collected patient-level information on diagnoses, signs/symptoms and tests. Study inclusion criteria were: ages 18 years or over at cancer diagnosis, no previous cancer, minimum 1 year of CPRD records pre-cancer, meeting CPRD quality criteria. CPRD covers 6.9% of the UK population and is representative of the general population [30], and as expected by the proportion of national coverage, 6.5% of all incident colon cancers registered in England during the study period were linked to active and up-to-standard CPRD records (*N* = 6316 out of 97,937 colon cancers diagnosed 2005–2010). After excluding patients with missing socio-demographic or route to diagnosis, *N* = 5745 individuals were included. Further details are reported elsewhere [8, 31].

### Study variables

The study outcome was emergency cancer diagnosis, defined according to the Routes to Diagnosis algorithm, i.e. diagnosis following presentation to Accident and Emergency, GP emergency referrals, or emergency pathways for in/out-patients [6, 32]. Non-emergency diagnoses included routine GP referrals, two-week wait referral, inpatient/outpatient elective and screening.

The main explanatory variables were comorbidities recorded before the diagnosis of cancer. Referring to the literature [17] and clinical experts we compiled a list of comorbidities potentially influencing the colon cancer diagnosis through different mechanisms: 1) ‘serious’ or complex comorbidities diagnosed/treated in secondary care, which can interfere with the cancer diagnosis through competing demands: e.g. cardiac, chronic respiratory, neurological diseases; 2) comorbidities possibly providing alternative explanations for signs/symptoms of cancer: gastrointestinal (GI) conditions (irritable bowel syndrome (IBS), diverticular, coeliac, inflammatory bowel, other GI diseases), gynaecological conditions (endometriosis, dysmenorrhoea), anxiety/depression; 3) comorbidities potentially offering opportunities for earlier diagnosis through regular GP visits: hypertension monitoring. Some comorbidities might act through multiple mechanisms: e.g. anxiety/depression, gynaecological conditions or inflammatory bowel diseases might provide alternative explanations for cancer symptoms (abdominal pain, diarrhoea, constipation), and also interfere with the ability to focus on cancer symptoms through competing demands.

‘Serious’ or complex comorbidities (see ‘1’ above) were defined using relevant ICD-10- diagnosis codes in hospital care records (Hospital Episodes Statistics-HES) [33, 34] relating to hospital care episodes during a two-year period before the cancer diagnosis. As linked HES records were available from 2003 onwards, a two-year pre-diagnostic window was chosen allowing the same HES observation period for all patients. We also created a binary variable coded as one if any HES record of ‘serious’ non-GI comorbidity versus none, excluding GI-conditions to focus on the competing demand mechanism.

Comorbidities possibly providing alternative explanations and those offering opportunities for earlier diagnosis (see ‘2’ and ‘3’ above) were defined using relevant Medcodes/Readcodes in CPRD relating to consultations for up to 10-years before the diagnosis of cancer. Comorbidities possibly presenting with abdominal symptoms (GI and gynaecological conditions), thus providing alternative explanations, were categorized as ‘new onset’ (if first recorded in the pre-diagnostic year) and ‘chronic/past’ (if already recorded 2–10 years pre-cancer). We hypothesised that ‘new onset’ comorbidities might include mis-diagnoses (where cancer symptoms were mis-interpreted as benign conditions), rather than true comorbidities (chronic conditions not related to cancer). Due to sparse data, IBS and diverticular disease were grouped together.

Further explanatory variables were pre-diagnostic alarm symptoms/signs (rectal bleeding, change in bowel habit, anaemia) and other abdominal symptoms (e.g. abdominal pain, constipation, diarrhoea) [10, 35, 36]. As previously described [8] Medcodes/Readcodes for symptoms were applied to CPRD records in the 10 years pre-diagnosis. Socio-demographic characteristics included gender, age and deprivation (Index of Multiple Deprivation for England). Cancer sub-sites were classified into distal (left) colon (i.e. splenic flexure, descending colon, sigmoid colon) (ICD C18.5-C18.7) and proximal (right) colon (i.e. caecum, appendix, ascending colon, hepatic flexure, transverse) (C18.0-C18.4) [27–29].

### Statistical analysis

We compared comorbidities, signs/symptoms and socio-demographic characteristics among emergency versus non-emergency presenters. We examined whether consultation rates for cancer symptoms during the pre-diagnostic year varied by specific comorbidities, controlling for socio-demographic factors, using negative binomial regression. The Pearson goodness-of-fit and likelihood ratio tests comparing negative binomial with Poisson regression models, indicated that negative binomial regression was more appropriate due to over-dispersion.

We evaluated the proportion of emergency presenters with alarm symptoms recorded in the pre-diagnostic year by comorbidity status, to evaluate if opportunities for earlier diagnosis vary by comorbidity type. Using multivariable logistic regression we examined the risk of emergency presentations for different comorbidities, controlling for socio-demographic characteristics, GP consultations and signs/symptoms, cancer sub-site and year of cancer diagnosis. Random effects accounted for possible patient clustering by practice. Men and women were examined separately and we assessed effect modification by age. In line with previous research on colon cancer [37], we decided a priori to examine men and women separately, considering that comorbidities differ in men and women and their impact on emergency presentations might be modified by gender. In particular, gynaecological conditions only affect women and other chronic conditions, such as Irritable Bowel Syndrome (IBS), affect women much more frequently than men [38, 39]. Tumour factors also differ by gender: proximal cancers, which often present with non-specific symptoms and are beyond the reach of flexible sigmoidoscopy [40], occur more frequently in women, possibly leading to gender differences in diagnostic complexity.

Finally, in addition to using the traditional epidemiological methods described above, we analysed the same data also using potential-outcomes or counterfactual-based approaches, estimating the effects of comorbidities on the risk of cancer being diagnosed through emergency rather than non-emergency routes in the population of colon cancer patients. We considered that covariates (socio-demographic factors and symptoms) might have differential effects on the exposure (comorbidity) and also affect the outcome (emergency presentations) (see Additional file 1 for a graphic representation and methodological details).

Statistical analyses were performed using STATA14 (Stata Corp., College Station, TX, USA).

## Results

### Comorbidities recorded prior to cancer diagnosis among emergency or non-emergency presenters

Among the 5745 colon cancer patients, 34% of women and 30% of men were diagnosed as emergencies. Overall, emergency cancer diagnosis occurred in 43% of patients with pre-existing ‘serious’ non-GI comorbidity versus 27% in individuals without comorbidity (*p* < 0.001). Examining specific pre-existing ‘serious’ comorbidities has shown that almost all of them were more prevalent in emergency than non-emergency presenters (Table 1).

**Table 1:**
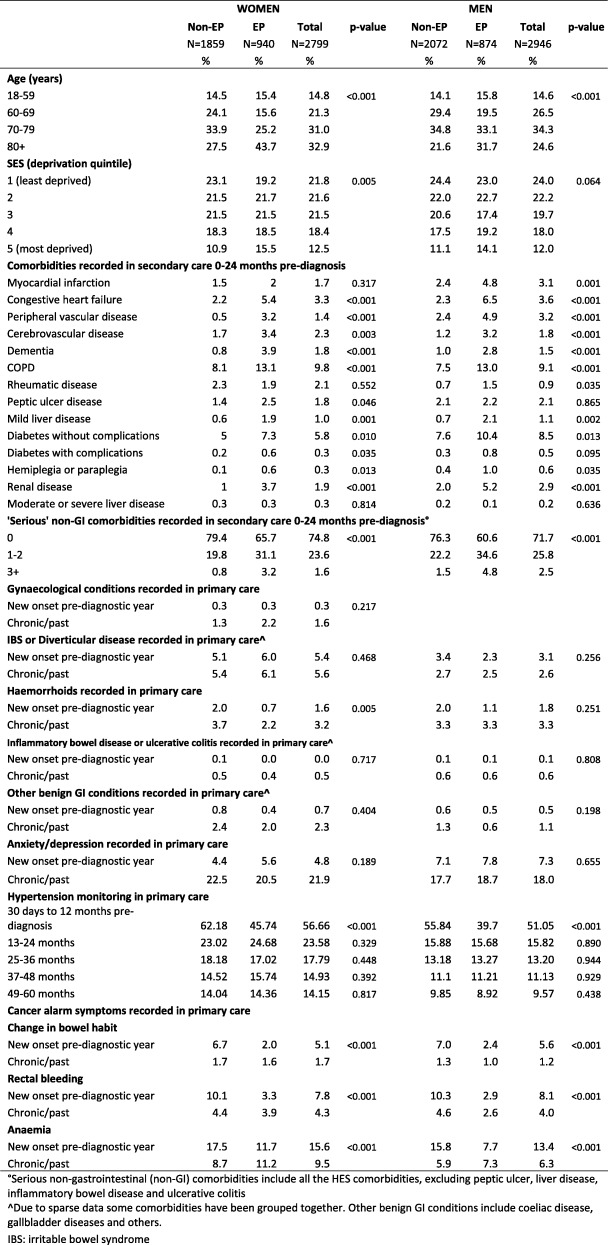
Socio-demographic characteristics, comorbidities and cancer alarm symptoms among individuals diagnosed with colon cancer following emergency (EP) and Non-emergency presentation (non-EP)

Primary care comorbidities were similar in emergency and non-emergency presenters, except for a higher frequency among non-emergency presenters of haemorrhoids in women and hypertension monitoring in both genders (Table 1). The most frequent primary care comorbidities were anxiety/depression and IBS/diverticular disease. IBS/diverticular diseases showed a stable prevalence 2–5 years pre-cancer (women: 1.4 to 2.3%; men: 0.5 to 1%), with a marked increase in the pre-diagnostic year (women: 6.2%; men: 3.5%) (data not shown).

### Consultations with potential cancer symptoms among comorbid and non-comorbid patients

**Fig. 1:**
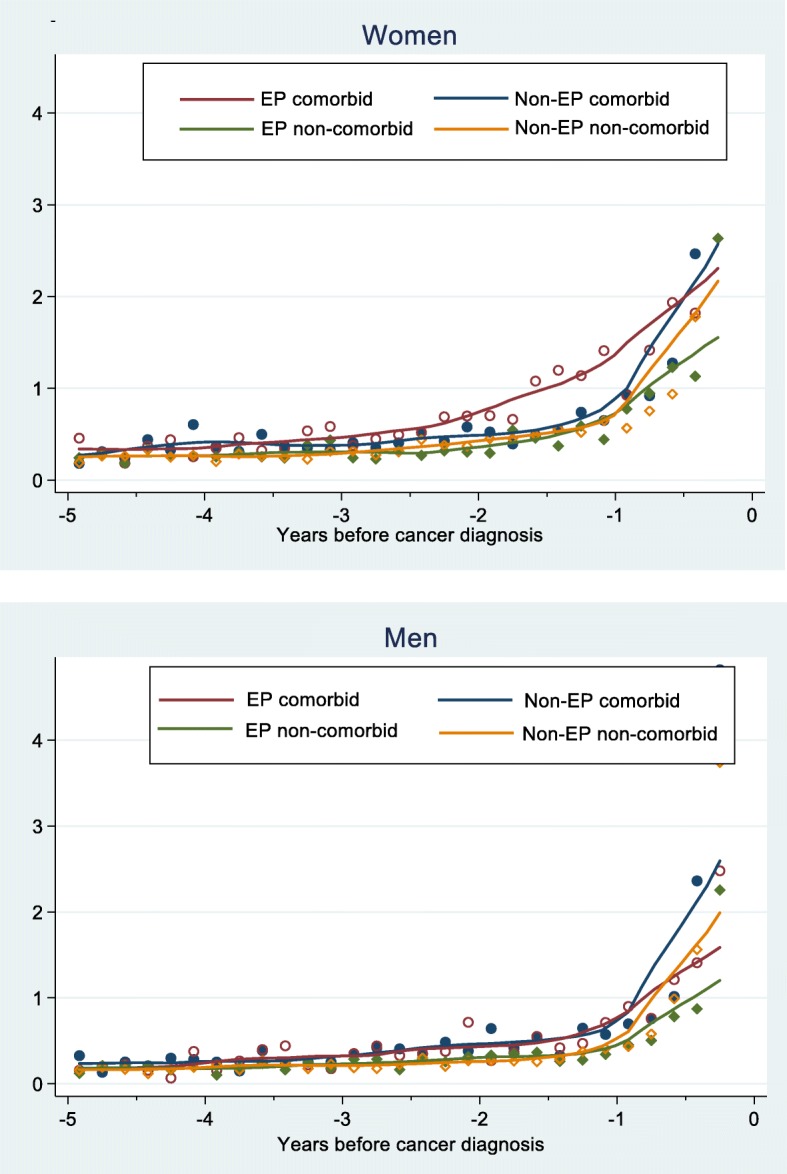
Consultation rates in primary care for cancer symptoms pre-diagnosis for emergency (EP) and non-emergency presenters (non-EP) with and without hospital-treated comorbidities. Observed data points and fitted local polynomial regression lines

**Fig. 2:**
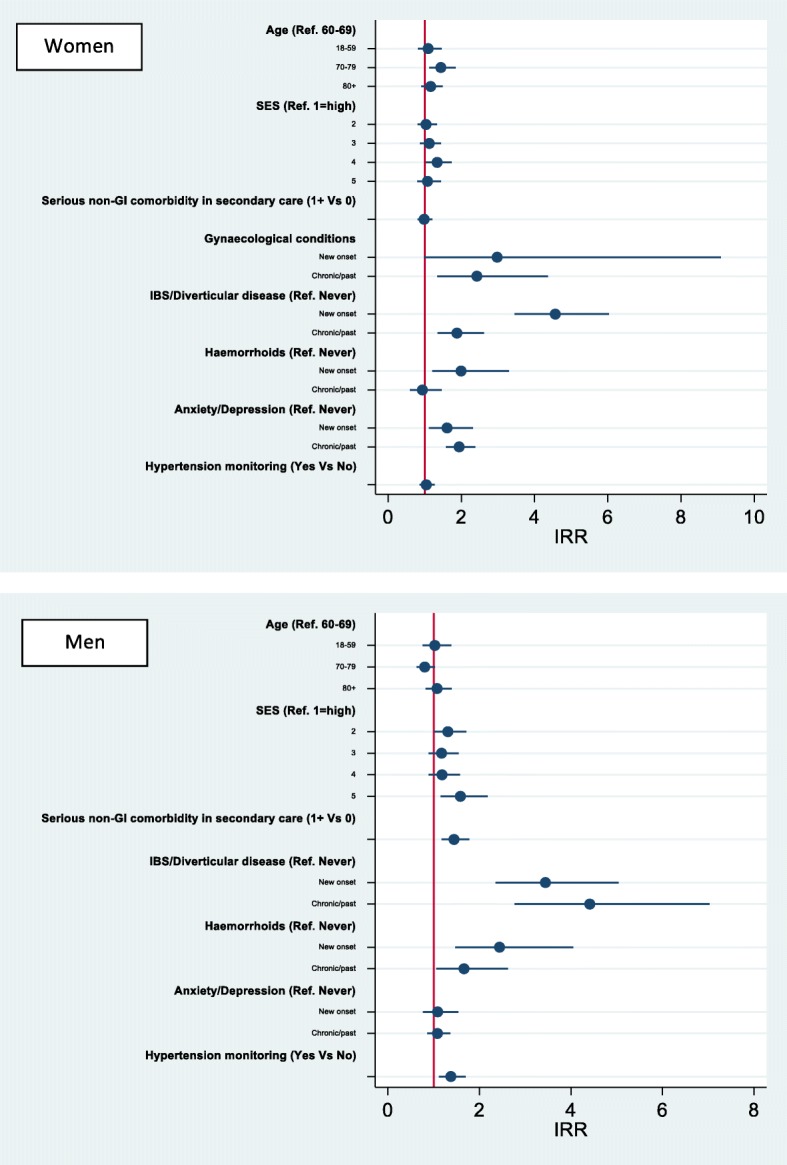
Incidence Rate Ratios (IRR) for primary care consultations with relevant symptoms during the pre-diagnostic year. Negative binomial regression including in the model all the variables shown in the figure, as well as cancer sub-site. SES=Socio-economic status; New onset comorbidity = comorbidity first recorded during the year pre-cancer diagnosis; Chronic/past = already recorded > 12 months pre-cancer diagnosis; Hypertension monitoring between 30 days and 12 months pre-cancer diagnosis

Consultation rates with potential cancer symptoms over the 5-years pre-diagnosis were stable up to the pre-diagnostic year, when a marked increase was observed for emergency and non-emergency presenters. However, among female emergency presenters with ‘serious’ non-GI comorbidities diagnosed/treated in secondary care, GP consultations with potential cancer symptoms started increasing 2 years pre-diagnosis (Fig. 1). Comorbid individuals consulted more frequently with cancer symptoms during the pre-diagnostic year than non-comorbid individuals, controlling for socio-demographic factors and cancer sub-site (Fig. 2). Among emergency presenters, the proportion of patients with cancer alarm symptoms (rectal bleeding, anaemia, change in bowel habit) recorded in primary care in the pre-diagnostic year (i.e. indicating possible missed diagnostic opportunities) was 21% among men with ‘serious’ hospital-treated comorbidities versus 11% among non-comorbid men (*p* < 0.001) (Table in Additional file 2). Patients with anxiety/depression also more frequently had alarm symptoms recorded the year before emergency cancer diagnosis compared to those never having had anxiety/depression (women: 36% versus 18% (*p* = 0.002); men 22% versus 13% (*p* = 0.021). Similarly, female emergency presenters with IBS/diverticular disease had more frequent records of alarm symptoms in the year pre-cancer diagnosis compared to those never having had IBS/diverticular disease (32% versus 19%; *p* = 0.008).

### Multivariable analysis examining the effect of comorbidities on emergency presentations

**Fig. 3:**
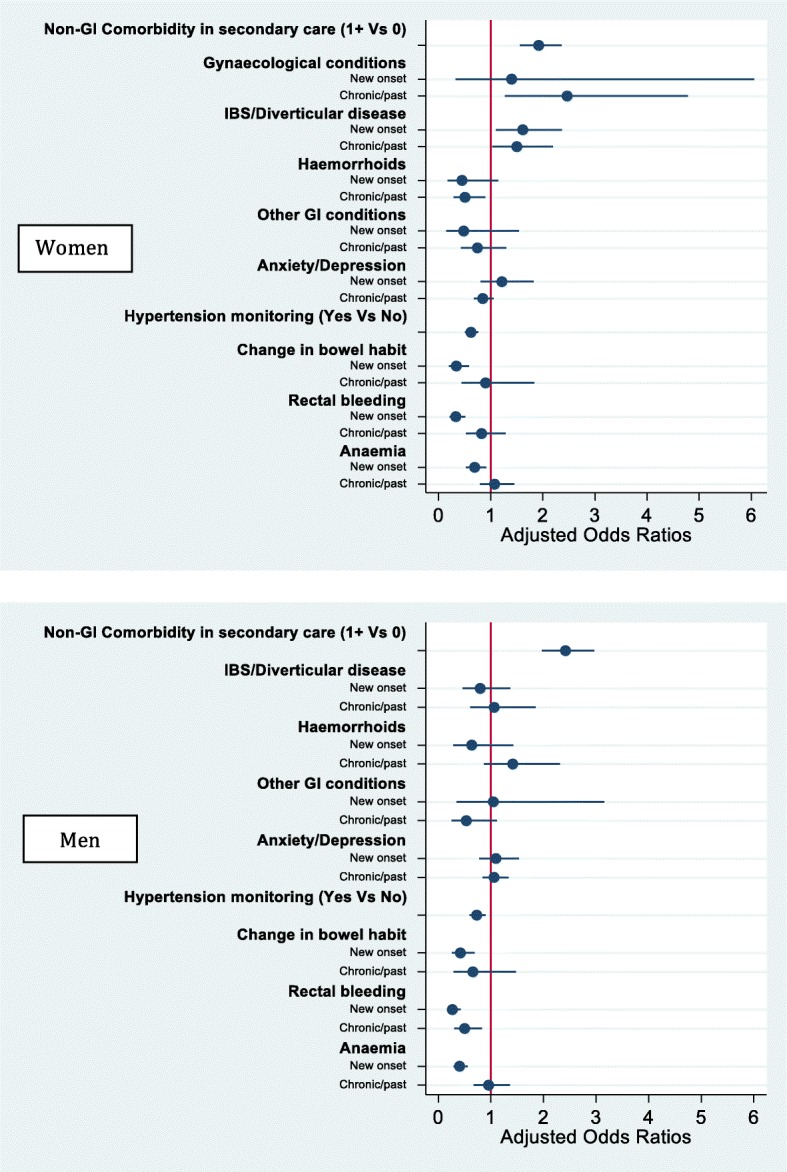
Likelihood of emergency colon cancer diagnosis: Mixed effects multivariable logistic regression Odds Ratios. Adjusted ORs from multivariable regression analysis including in the model all the variables shown in the figure, as well as socio-demographic characteristics and number of consultations in the pre-diagnostic year. New onset comorbidity = comorbidity first recorded during the year pre-cancer diagnosis; Chronic/past = comorbidity already recorded > 12 months pre-cancer diagnosis; Hypertension monitoring between 30 days and 12 months pre-cancer diagnosis

Men and women with pre-existing ‘serious’ non-GI comorbidities diagnosed/treated in secondary care had a significantly higher risk of emergency presentations, controlling for socio-demographic factors and symptoms (Fig. 3). In contrast, hypertension monitoring decreased the risk of emergency presentation. Among women, gynaecological and IBS/diverticular diseases increased the risk of emergency presentations. New onset alarm symptoms decreased emergency presentations for both genders.

Examining the effects of comorbidities stratified by age and controlling for deprivation, cancer sub-site and symptoms, showed how the risk of emergency presentation was particularly high for women aged less than 60 with gynaecological conditions (OR = 3.41; 95%CI 1.2–9.9) and for those with recent IBS/diverticular disease diagnoses (OR = 2.84; 95%CI 1.2–7.7) (Table 2). The risk was also higher for women aged 70–79 with gynaecological or ‘serious’ non-GI comorbidities, and for women aged 80 or more with a past history of IBS/diverticular disease or ‘serious’ non-GI comorbidities. For men, age-stratified results (data not shown) were similar to those for all age-groups combined shown in Fig. 3.

**Table 2:**
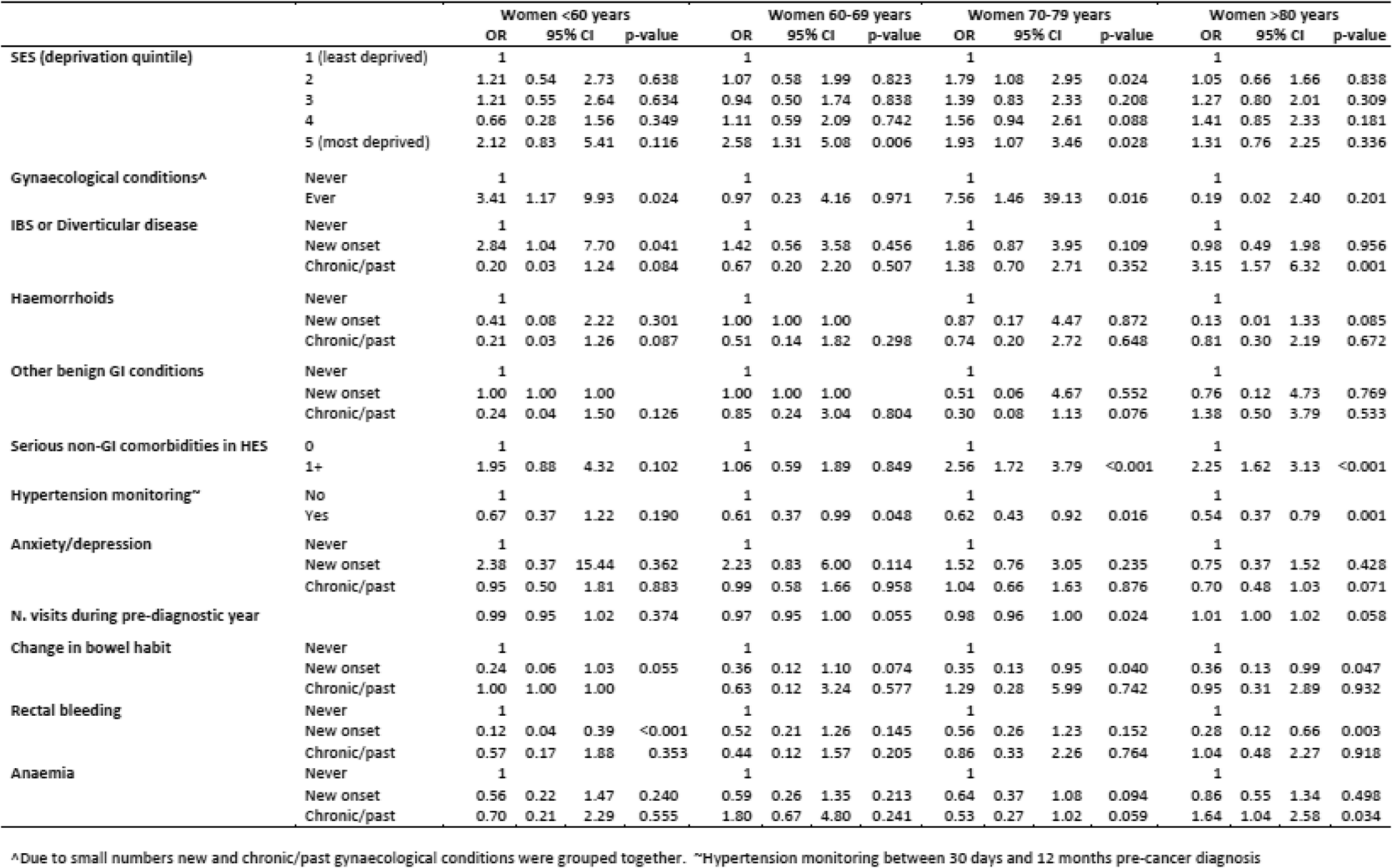
Age-stratified multivariable logistic regression Odds Ratios (OR) for the risk of emergency colon cancer diagnosis among women

#### Potential-outcomes approach estimating the average effect of comorbidities on emergency presentations

The findings obtained through standard multivariable analysis were corroborated using potential-outcomes methods (Table 3). Overall, at population level, the risk of emergency presentation for both genders was increased by ‘serious’ non-GI comorbidities. Age-stratified analyses showed a particularly high average risk for some subgroups of colon cancer patients, such as women aged less than 60 with a recent IBS/diverticular disease or benign gynaecological diagnosis, and women aged 80 or more with a past history of IBS/diverticular disease. All estimates accounted for socio-demographic characteristics, cancer symptoms and other comorbidities. In particular, Table 3 shows the potential outcome mean (POmean), which corresponds to the proportion of emergency cancer diagnoses we would expect among colon cancer patients if nobody had the examined comorbidity. After estimating the potential outcome mean we would expect if everybody had that comorbidity, we calculated the difference between the two means (risk difference or average treatment effect, ATE). The ATE corresponds to the average effect of each comorbidity on the risk of emergency presentations. For example, examining the effect of ‘serious’ non-GI comorbidities (recorded in HES) we obtained a POmean = 0.30 for women and POmean = 0.25 for men, indicating that in the *absence* of ‘serious’ non-GI comorbidities, 30% of women with colon cancer and 25% of men can be expected to have an emergency presentation. An ATE = 0.12 for women and ATE = 0.15 for men indicate that the *presence* of a ‘serious’ comorbidity would significantly increase these proportions (*p* < 0.001), adding a further 12 and 15% of emergency presentations among women and men, respectively. Overall, this would result in 42% of comorbid women with colon cancer and 40% of comorbid men having an emergency presentation. Significant effects were also found for COPD, diabetes and cardiac disease.

**Table 3:**
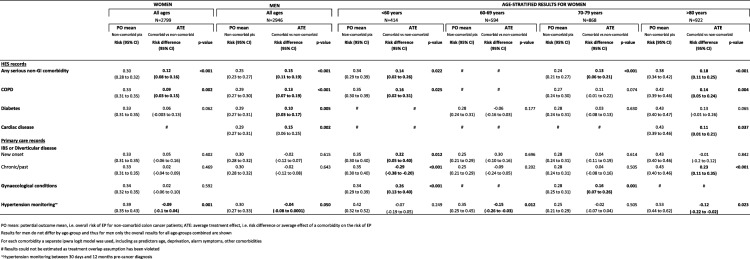
Estimated average effect (ATE) of comorbidities on emergency presentations and potential outcome (PO) mean, taking into account socio-demographic characteristics, other comorbidities and cancer alarm symptoms

In contrast, hypertension monitoring in primary care during the pre-diagnostic year had an average protective effect, albeit small, in women and men (ATE = -9% and ATE = -4%). The other comorbidities recorded in primary care had no significant effect when analysing all age-groups together.

Age-stratified analyses have shown that if women aged less than 60 never have a diagnosis of IBS/diverticular disease, the average risk of emergency presentation can be expected to be 35%. New onset IBS significantly increased emergency presentations (ATE = 22%), while chronic/past IBS decreased the risk (ATE = -29%). Thus, the overall risk of emergency presentations for women aged less than 60 with colon cancer and new onset IBS/diverticular disease would be 57%, while it would be 6% for those with a chronic/past IBS. Gynaecological conditions also significantly increased the risk for women aged less than 60. Among women aged 80 or more the risk of emergency diagnosis was also significantly increased by COPD or cardiac diseases or a past history of IBS/diverticular disease.

Findings for men did not differ by age-group (data not shown) and thus age-stratified results are only reported for women in Table 3.

## Discussion

### Summary

Most comorbidities increased the risk of emergency colon cancer diagnosis, but the effects are complex and vary by socio-demographic factors and by type and timing of comorbidity onset. Comorbid patients consulted more frequently with cancer symptoms than non-comorbid individuals in the pre-diagnostic year and some comorbidities offered opportunities for earlier diagnosis. The risk of emergency presentations was particularly high for some subgroups, including patients with ‘serious’ comorbidities diagnosed/treated in secondary care (diabetes, cardiac, respiratory diseases) and women aged less than 60 with a recent diagnosis of IBS/diverticular disease or benign gynaecological conditions. One-fifth of emergency presenters with ‘serious’ comorbidities, diagnosed/treated in secondary care, presented with cancer alarm symptoms in primary care during the pre-diagnostic year, suggesting opportunities for reducing emergency presentations. One in three female emergency presenters with a recent diagnosis of IBS/diverticular disease consulted their GP with cancer alarm symptoms in the year pre-cancer diagnosis.

Traditional epidemiological methods and counterfactual-based approaches yielded similar findings.

### Strengths and limitations

A detailed examination of specific comorbidities prospectively recorded in primary and secondary care allowed us to substantially add to the literature, highlighting how comorbidities can influence the risk of emergency presentations, acting through different mechanisms, with patients’ gender and age modifying their effect. It is the first population-based study, using high-quality primary and secondary care data linked to cancer registration and routes to diagnosis data, showing contrasting effects of different comorbidities on emergency presentations. By identifying higher risk groups the study can help develop targeted strategies for reducing emergency presentations. A substantial proportion of emergency presenters had primary care consultations with alarm symptoms during the pre-diagnostic year, particularly among comorbid patients, suggesting that there are opportunities for earlier diagnosis. Sensitivity analyses changing the time-window for defining ‘serious’ comorbidities diagnosed/treated in hospital (e.g. including only comorbidities recorded more than 6 or 12 months pre-cancer) confirmed an increased risk of emergency presentation for comorbid patients (data not shown).

Relying on routinely collected data we probably underestimated the prevalence of symptoms and comorbidities, but this likely occurred in a non-differential way, as information was prospectively recorded.

Linked data was only available for our analyses up to 2010. Emergency presentations for colorectal cancer have decreased in England between 2006 and 2010 (from 27 to 24%), however no further reductions have been reported since 2010 [2, 6]. Inequalities in emergency presentations [2] and poorer survival for cancer patients diagnosed as an emergency [41] are persisting problems. Similarly, in our study focusing specifically on colon cancer, emergency presentations decreased between 2006 and 2010 (from 36 to 32% among women and from 33 to 29% among men), but year of diagnosis did not modify the effect of comorbidities on emergency presentations (data not shown).

### Comparison with existing literature

The relationship between comorbidities and emergency presentations is complex, with multiple pathways possibly leading to delays (influenced by biological, psychological and organizational factors affecting patients and doctors) [1, 13, 42–45]. Recent research reported how ‘competing demands’ (for example, cardiac conditions) and ‘alternative explanation’ comorbidities (including IBS/diverticular diseases) are associated with longer diagnostic intervals [17], but their effects on emergency presentations were unknown.

In contrast, some previous studies [18, 42] reported how chronic ‘risk factor’ conditions, such as hypertension, which in themselves are typically asymptomatic, can offer opportunities for patients to mention possible cancer symptoms during regular healthcare encounters or for doctors to notice new sign/symptoms, leading to earlier cancer diagnosis, which is in line with our findings. While such ‘risk factor’ conditions, monitored in primary care, are usually not perceived as particularly worrying, this is not the case for ‘competing demands’ comorbidities, such as cardiac conditions or complicated diabetes treated in secondary care. ‘Competing demands’ comorbidities are frequently ‘serious’/complex to manage or require urgent clinical attention- and critically they can give rise to new symptoms. Thus, despite being associated with frequent healthcare encounters, instead of providing opportunities for earlier cancer diagnosis, ‘competing demands’ comorbidities are more likely to interfere with timely investigations of possible cancer symptoms, particularly if symptoms are vague [44, 46, 47], increasing the risk of emergency presentations.

Other chronic conditions, such as IBS or diverticular disease, can lead to delays by providing alternative explanations, influencing patients’ help-seeking for possible cancer symptoms and/or referrals for investigations. Sparse data limited the possibility of analysing IBS and diverticular diseases separately in our study. However, as IBS and diverticular diseases have many overlapping clinical features and often present with recurrent abdominal symptoms [48], they can both complicate differential diagnosis with cancer [49]. A Dutch study [50] reported how sometimes doctors attribute colorectal cancer symptoms to pre-existing conditions in patients with a history of diverticulitis or gynaecological conditions. Similarly, the higher risk of emergency cancer diagnosis among women aged less than 60 with a recent diagnosis of IBS/diverticular disease or benign gynaecological conditions highlighted in our study, can be explained at least in part by a mis-attribution of cancer symptoms to benign conditions. This is supported by our findings showing a marked increase in IBS/diverticular disease records shortly before the cancer diagnosis. The ‘baseline’ prevalence of IBS/diverticular disease 2–5 years pre-cancer in our sample was low and similar to previous primary care studies (2.5%) [51, 52]. IBS prevalence in the general population varies widely [52], depending on diagnostic criteria and data sources [39], but what is noteworthy here is the increase during the pre-diagnostic months. Some cases might have been ‘working’ diagnoses possibly followed by investigations, nevertheless our study highlighted how a recent IBS diagnosis can increase emergency presentations particularly in women.

In contrast, long-standing IBS/diverticular diseases had a protective effect in younger women, suggesting that in these circumstances women and/or doctors recognized that symptoms had changed. Familiarity with the healthcare system, more opportunities to discuss symptoms with the doctor and tumour biology might also have played a role, with some women with long-standing symptoms possibly having slow-growing and less aggressive cancers. Differently from younger women, older women with a past history of IBS/diverticular disease had an increased risk of emergency presentations. Advanced age might have prevented invasive investigations due to patients’ health status, their preferences or barriers accessing healthcare [53].

Patients sometimes attribute cancer symptoms to comorbidities or delay reporting them due to worries about wasting doctors’ time [45, 54]. We could not examine cancer awareness or timely reporting of symptoms, however our study highlighted how comorbid patients consulted significantly more with cancer symptoms than non-comorbid individuals. Thus, diagnostic delays cannot simply be explained by patients not seeing their doctor for cancer symptoms.

### Implications for research and practice

One in three colon cancers are diagnosed as an emergency, with higher risks for comorbid patients especially if belonging to the youngest or oldest age-groups. Reducing emergency cancer diagnosis is an important public health target, given its negative consequences in terms of survival, independently of stage [1, 3]. Appropriate interventions are necessary for the large number of individuals with comorbidities who experience potential cancer symptoms. Innovative diagnostic strategies need to be developed targeting higher risk groups and taking into account the specific mechanisms through which comorbidities might affect diagnostic timeliness. In particular, greater integration between primary and secondary care, as well as more extensive use of multi-disciplinary diagnostic centres, which are currently being evaluated in different countries [55–57], can play an important role for complex patients, such as those with comorbidities. Sufficient consultation time is important especially for patients with multi-morbidities and vague symptoms in order to adopt a holistic approach and reduce delays [58]. Optimization of healthcare services [59–61] and support from nurses could help to free up consultation time. In addition to system-wide approaches, targeted interventions are needed. For example, colon cancer diagnosis in women can be particularly complex because of gynaecological conditions and the higher prevalence of IBS [38, 39], highlighting the importance of safety-netting strategies and innovative diagnostic approaches tailored for higher risk groups. Recent research [62, 63] and NICE guidelines [36], indicate that quantitative faecal haemoglobin testing (FIT) can be useful for patients presenting with abdominal symptoms in primary care to identify those who might benefit from further investigations. Moreover, according to international experts, a colonoscopy is indicated for patients aged 50 years and over with symptoms such as diarrhoea and mixed bowel habit [64, 65]. Relatedly, the American Gastroenterology Association recently recommended excluding colon cancer with modern techniques and colonoscopy after the first episode of diverticulitis [49].

International evidence on emergency cancer diagnosis is scant, but what there is suggests that the problem is not limited to the UK [1], particularly for cancers initially presenting with non-specific symptoms. More international data could provide insights into the role played by healthcare factors in influencing diagnostic timeliness. Patient’s healthcare records can be a useful resource not only for reporting composite comorbidity measures, such as the Charlson comorbidity index, but also to evaluate specific comorbidities. The importance of examining specific comorbidities is increasingly being recognised in the international literature [44, 47, 66, 67]. A detailed analysis of type and timing of comorbidities and specific pathways is necessary to better understand the mechanisms leading to delays and identify appropriate interventions. The effects of comorbidities are complex and patient, doctors and healthcare system factors all need to be considered in order to reduce their impact on emergency presentations and improve cancer outcomes.

## Conclusions

The study highlighted how most comorbidities increased the risk of emergency colon cancer diagnosis, but the effects are complex and vary by socio-demographic factors and by type of comorbidity. The risk of emergency presentation was particularly high for some subgroups, including patients with ‘serious’ comorbidities diagnosed/treated in hospital during the years pre-cancer diagnosis (diabetes, cardiac, respiratory diseases) and women aged less than 60 with a recent diagnosis of IBS/diverticular disease or benign gynaecological conditions. By identifying higher risk groups the study can help develop targeted strategies for reducing emergency presentations. A substantial proportion of emergency presenters had primary care consultations with alarm symptoms during the pre-diagnostic year, particularly among comorbid patients. This suggests that comorbidities may interfere with diagnostic reasoning or investigations due to ‘competing demands’ or because they provide ‘alternative explanations’. In contrast, the management of chronic risk factors such as hypertension may offer opportunities for earlier diagnosis. Interventions are needed to support the diagnostic process in comorbid patients. Appropriate guidelines and diagnostic services to support the evaluation of new or changing symptoms in comorbid patients may be useful.

## Additional files

Additional file 1: Pathways linking comorbidities to emergency presentations and potential-outcomes methods. Graphic representation and details on the potential-outcomes methods. (PDF 221 kb)

Additional file 2: Prevalence of cancer alarm symptoms in the pre-diagnostic year among comorbid and non-comorbid patients diagnosed with colon cancer following emergency presentation (EP) (PDF 51 kb)

## Abbreviations

ATE: Average treatment effect
CI: Confidence interval
COPD: Chronic obstructive pulmonary disease
CPRD: Clinical practice research datalink
EP: Emergency presentations
GI: Gastrointestinal
HES: Hospital Episode Statistics
IBS: Irritable bowel syndrome
OR: Odds ratio
POmean: Potential outcome mean

## References

[1] Zhou Y, Abel GA, Hamilton W, Pritchard-Jones K, Gross CP, Walter FM, Renzi C, Johnson S, McPhail S, Elliss-Brookes L (2017). Diagnosis of cancer as an emergency: a critical review of current evidence. Nat Rev Clin Oncol.

[2] Abel GA, Shelton J, Johnson S, Elliss-Brookes L, Lyratzopoulos G (2015). Cancer-specific variation in emergency presentation by sex, age and deprivation across 27 common and rarer cancers. Br J Cancer.

[3] Independent Cancer Taskforce: Achieving world-class cancer outcomes: a strategy for England 2015–2020.2015 Available at http://www.cancerresearchuk.org/sites/default/files/achieving_world-class_cancer_outcomes_-_a_strategy_for_england_2015-2020.pdf. Accessed 1 Mar 2016.

[4] Salika T, Abel GA, Mendonca SC, von Wagner C, Renzi C, Herbert A, McPhail S, Lyratzopoulos G. Associations between diagnostic pathways and care experience in colorectal cancer: evidence from patient-reported data. Frontline Gastroenterology. 2018;9(3):241–48. 10.1136/flgastro-2017-100926 PMC6056077 30046429

[5] Goodyear SJ, Leung E, Menon A, Pedamallu S, Williams N, Wong LS (2008). The effects of population-based faecal occult blood test screening upon emergency colorectal cancer admissions in Coventry and north Warwickshire. Gut.

[6] Routes to diagnosis 2006-2013 workbook. Public Health England. Available at http://www.ncin.org.uk/publications/routes_to_diagnosishttp://www.ncin.org.uk/publications/routes_to_diagnosis. Accessed 1 Aug 2018.

[7] Lyratzopoulos G, Saunders CL, Abel GA (2014). Are emergency diagnoses of cancer avoidable? A proposed taxonomy to motivate study design and support service improvement. Future Oncol.

[8] Renzi C, Lyratzopoulos G, Card T, Chu TP, Macleod U, Rachet B (2016). Do colorectal cancer patients diagnosed as an emergency differ from non-emergency patients in their consultation patterns and symptoms? A longitudinal data-linkage study in England. Br J Cancer.

[9] Black G, Sheringham J, Spencer-Hughes V, Ridge M, Lyons M, Williams C, Fulop N, Pritchard-Jones K (2015). Patients’ experiences of Cancer diagnosis as a result of an emergency presentation: a qualitative study. PLoS One.

[10] Sheringham JR, Georghiou T, Chitnis XA, Bardsley M (2014). Comparing primary and secondary health-care use between diagnostic routes before a colorectal cancer diagnosis: cohort study using linked data. Br J Cancer.

[11] Gunnarsson H, Jennische K, Forssell S, Granstrom J, Jestin P, Ekholm A, Olsson LI (2014). Heterogeneity of Colon Cancer patients reported as emergencies. World J Surg.

[12] Abel GA, Mendonca SC, McPhail S, Zhou Y, Elliss-Brookes L, Lyratzopoulos G (2017). Emergency diagnosis of cancer and previous general practice consultations: insights from linked patient survey data. Br J Gen Pract.

[13] Wallace D, Walker K, Kuryba A, Finan P, Scott N, van der Meulen J (2014). Identifying patients at risk of emergency admission for colorectal cancer. Br J Cancer.

[14] Department of Health. Report. Long-term conditions compendium of Information. 3rd ed; 2012. https://www.gov.uk/government/uploads/system/uploads/attachment_data/file/216528/dh_134486.pdf. Accessed 10 Mar 2018.

[15] Mujica-Mota RE, Roberts M, Abel G, Elliott M, Lyratzopoulos G, Roland M, Campbell J (2015). Common patterns of morbidity and multi-morbidity and their impact on health-related quality of life: evidence from a national survey. Qual Life Res.

[16] Barnett K, Mercer SW, Norbury M, Watt G, Wyke S, Guthrie B (2012). Epidemiology of multimorbidity and implications for health care, research, and medical education: a cross-sectional study. Lancet.

[17] Mounce LTA, Price S, Valderas JM, Hamilton W (2017). Comorbid conditions delay diagnosis of colorectal cancer: a cohort study using electronic primary care records. Br J Cancer.

[18] Salika T, Lyratzopoulos G, Whitaker KL, Waller J, Renzi C. Do comorbidities influence help-seeking for cancer alarm symptoms? A population-based survey in England. J Public Health. 2017;24:1–10. 10.1093/pubmed/fdx072 PMC6105929 28655212

[19] Lesko CR, Buchanan AL, Westreich D, Edwards JK, Hudgens MG, Cole SR (2017). Generalizing study results: a potential outcomes perspective. Epidimiology.

[20] Anothaisintawee T, Udomsubpayakul U, McEvoy M, Lerdsitthichai P, Attia J, Thakkinstian A (2016). Effect of lipophilic and hydrophilic statins on breast Cancer risk in Thai women: a cross-sectional study. J Cancer.

[21] Hernan MA, and Robins J: Causal Inference 2017.(Last accessed Jan 2018) Available at www.hsph.harvard.edu/miguel-hernan/causal-inference. Accessed 15 Jan 2018.

[22] Glass TA, Goodman SN, Hernan MA, Samet JM (2013). Causal inference in public health. Annu Rev Public Health.

[23] Zigler CM, Dominici F (2014). Point: clarifying policy evidence with potential-outcomes thinking--beyond exposure-response estimation in air pollution epidemiology. Am J Epidemiol.

[24] Glymour MM, Spiegelman D (2017). Evaluating public health interventions: 5. Causal inference in public Health Research-do sex, race, and biological factors cause health outcomes?. Am J Public Health.

[25] Rehkopf DH, Glymour MM, Osypuk TL (2016). The consistency assumption for causal inference in social epidemiology: when a rose is not a rose. Curr Epidemiol Rep.

[26] Hernan MA (2016). Does water kill? A call for less casual causal inferences. Ann Epidemiol.

[27] Doubeni CA, Corley DA, Quinn VP, Jensen CD, Zauber AG, Goodman M, Johnson JR, Mehta SJ, Becerra TA, Zhao WK, et al. Effectiveness of screening colonoscopy in reducing the risk of death from right and left colon cancer: a large community-based study. Gut. 2018;67(2):291–98. 10.1136/gutjnl-2016-312712 PMC5868294 27733426

[28] Karim S, Brennan K, Nanji S, Berry SR, Booth CM. Association between prognosis and tumor laterality in early-stage Colon Cancer. JAMA Oncol. 2017;3(10):1386–92. 10.1001/jamaoncol.2017.1016 PMC5710504 28594974

[29] Hansen PL, Hjertholm P, Vedsted P (2015). Increased diagnostic activity in general practice during the year preceding colorectal cancer diagnosis. Int J Cancer.

[30] Herrett E, Gallagher AM, Bhaskaran K, Forbes H, Mathur R, van Staa T, Smeeth L (2015). Data resource profile: clinical practice research datalink (CPRD). Int J Epidemiol.

[31] Renzi C, Lyratzopoulos G, Hamilton W, Rachet B. Opportunities for reducing emergency diagnoses of colon cancer in women and men: a data-linkage study on pre-diagnostic symptomatic presentations and benign diagnoses. Eur J Cancer Care. 2019. p. e13000. 10.1111/ecc.13000. 10.1111/ecc.13000 PMC6492167 30734381

[32] Elliss-Brookes L, McPhail S, Ives A, Greenslade M, Shelton J, Hiom S, Richards M (2012). Routes to diagnosis for cancer - determining the patient journey using multiple routine data sets. Br J Cancer.

[33] Shack LG, Rachet B, Williams EM, Northover JM, Coleman MP (2010). Does the timing of comorbidity affect colorectal cancer survival? A population based study. Postgrad Med J.

[34] Maringe C, Fowler H, Rachet B, Luque-Fernandez MA (2017). Reproducibility, reliability and validity of population-based administrative health data for the assessment of cancer non-related comorbidities. PLoS One.

[35] Din NU, Ukoumunne OC, Rubin G, Hamilton W, Carter B, Stapley S, Neal RD (2015). Age and gender variations in Cancer diagnostic intervals in 15 cancers: analysis of data from the UK clinical practice research datalink. PLoS One.

[36] NICE guidelines [NG12]: Suspected cancer: recognition and referral. Available at http://www.nice.org.uk/guidance/NG12/chapter/1-Recommendations-organised-by-site-of-cancer. Accessed 3 Oct 2017.

[37] Fowler H, Belot A, Njagi EN, Luque-Fernandez MA, Maringe C, Quaresma M, Kajiwara M, Rachet B (2017). Persistent inequalities in 90-day colon cancer mortality: an English cohort study. Br J Cancer.

[38] Lovell RM, Ford AC (2012). Global prevalence of and risk factors for irritable bowel syndrome: a meta-analysis. Clin Gastroenterol Hepatol.

[39] Sperber AD, Dumitrascu D, Fukudo S, Gerson C, Ghoshal UC, Gwee KA, Hungin APS, Kang JY, Minhu C, Schmulson M (2017). The global prevalence of IBS in adults remains elusive due to the heterogeneity of studies: a Rome Foundation working team literature review. Gut.

[40] Holme O, Schoen RE, Senore C, Segnan N, Hoff G, Loberg M, Bretthauer M, Adami HO, Kalager M (2017). Effectiveness of flexible sigmoidoscopy screening in men and women and different age groups: pooled analysis of randomised trials. BMJ (Clin Res Ed).

[41] Exarchakou A, Rachet B, Belot A, Maringe C, Coleman MP (2018). Impact of national cancer policies on cancer survival trends and socioeconomic inequalities in England, 1996-2013: population based study. BMJ (Clin Res Ed).

[42] Gunnarsson H, Holm T, Ekholm A, Olsson LI (2011). Emergency presentation of colon cancer is most frequent during summer. Color Dis.

[43] Mitchell E, Pickwell-Smith B, Macleod U (2015). Risk factors for emergency presentation with lung and colorectal cancers: a systematic review. BMJ Open.

[44] Gurney J, Sarfati D, Stanley J (2015). The impact of patient comorbidity on cancer stage at diagnosis. Br J Cancer.

[45] McLachlan S, Mansell G, Sanders T, Yardley S, van der Windt D, Brindle L, Chew-Graham C, Little P (2015). Symptom perceptions and help-seeking behaviour prior to lung and colorectal cancer diagnoses: a qualitative study. Fam Pract.

[46] Gurney J, Sarfati D, Stanley J, Dennett E, Johnson C, Koea J, Simpson A, Studd R (2013). Unstaged cancer in a population-based registry: prevalence, predictors and patient prognosis. Cancer Epidemiol.

[47] Corkum M, Urquhart R, Kendell C, Burge F, Porter G, Johnston G (2012). Impact of comorbidity and healthcare utilization on colorectal cancer stage at diagnosis: literature review. Cancer Causes Control.

[48] Strate LL, Modi R, Cohen E, Spiegel BM (2012). Diverticular disease as a chronic illness: evolving epidemiologic and clinical insights. Am J Gastroenterol.

[49] Regula J (2016). Diverticular disease and colorectal Cancer: incidental diagnosis or real association? Final answer. J Clin Gastroenterol.

[50] Brandenbarg D, Groenhof F, Siewers IM, van der Voort A, Walter FM, Berendsen AJ (2018). Possible missed opportunities for diagnosing colorectal cancer in Dutch primary care: a multimethods approach. Br J Gen Pract.

[51] Hamilton W, Lancashire R, Sharp D, Peters TJ, Cheng K, Marshall T (2009). The risk of colorectal cancer with symptoms at different ages and between the sexes: a case-control study. BMC Med.

[52] Thompson WG, Heaton KW, Smyth GT, Smyth C (2000). Irritable bowel syndrome in general practice: prevalence, characteristics, and referral. Gut.

[53] Tate AR, Nicholson A, Cassell JA (2010). Are GPs under-investigating older patients presenting with symptoms of ovarian cancer? Observational study using general practice research database. Br J Cancer.

[54] Hall N, Birt L, Banks J, Emery J, Mills K, Johnson M, Rubin GP, Hamilton W, Walter FM (2015). Symptom appraisal and healthcare-seeking for symptoms suggestive of colorectal cancer: a qualitative study. BMJ Open.

[55] Moseholm E, Lindhardt BO (2017). Patient characteristics and cancer prevalence in the Danish cancer patient pathway for patients with serious non-specific symptoms and signs of cancer-a nationwide, population-based cohort study. Cancer Epidemiol.

[56] Naeser E, Fredberg U, Moller H, Vedsted P (2017). Clinical characteristics and risk of serious disease in patients referred to a diagnostic Centre: a cohort study. Cancer Epidemiol.

[57] Nicholson BD, Oke J, Friedemann Smith C, Phillips JA, Lee J, Abel L, Kelly S, Gould I, Mackay T, Kaveney Z (2018). The suspected CANcer (SCAN) pathway: protocol for evaluating a new standard of care for patients with non-specific symptoms of cancer. BMJ Open.

[58] Mitchell E, Rubin G, Merriman L, Macleod U (2015). The role of primary care in cancer diagnosis via emergency presentation: qualitative synthesis of significant event reports. Br J Cancer.

[59] Hobbs FDR, Bankhead C, Mukhtar T, Stevens S, Perera-Salazar R, Holt T, Salisbury C (2016). Clinical workload in UK primary care: a retrospective analysis of 100 million consultations in England, 2007-14. Lancet.

[60] BMA (2016). Safe working in general practice.

[61] Rimmer A (2017). Give GPs more time with patients, urges RCGP chair. BMJ (Clin Res Ed).

[62] Hogberg C, Karling P, Rutegard J, Lilja M (2017). Diagnosing colorectal cancer and inflammatory bowel disease in primary care: the usefulness of tests for faecal haemoglobin, faecal calprotectin, anaemia and iron deficiency. A prospective study. Scand J Gastroenterol.

[63] Mowat C, Digby J, Strachan JA, Wilson R, Carey FA, Fraser CG, Steele RJ (2016). Faecal haemoglobin and faecal calprotectin as indicators of bowel disease in patients presenting to primary care with bowel symptoms. Gut.

[64] Moayyedi P, Mearin F, Azpiroz F, Andresen V, Barbara G, Corsetti M, Emmanuel A, Hungin APS, Layer P, Stanghellini V (2017). Irritable bowel syndrome diagnosis and management: a simplified algorithm for clinical practice. United European Gastroenterol J.

[65] Ford AC, Lacy BE, Talley NJ (2017). Irritable bowel syndrome. N Engl J Med.

[66] Geraci JM, Escalante CP, Freeman JL, Goodwin JS (2005). Comorbid disease and cancer: the need for more relevant conceptual models in health services research. J Clin Oncol.

[67] Fleming ST, Pursley HG, Newman B, Pavlov D, Chen K (2005). Comorbidity as a predictor of stage of illness for patients with breast cancer. Med Care.

