# Supplementary material for: Contrasting effects of comorbidities on emergency colon cancer diagnosis: a longitudinal data-linkage study in England
Source: BMC Health Serv Res. 2019 May 15;19:311. doi: 10.1186/s12913-019-4075-4 (PMC6521448; doi:10.1186/s12913-019-4075-4)
Supplement: Supplementary file 1 — Pathways linking comorbidities to emergency presentations and potential-outcomes methods. Graphic representation and details on the potential-outcomes methods. (PDF 221 kb) [file 12913_2019_4075_MOESM1_ESM.pdf]

Figure: Pathways linking comorbidities to emergency presentations and relevant covariates

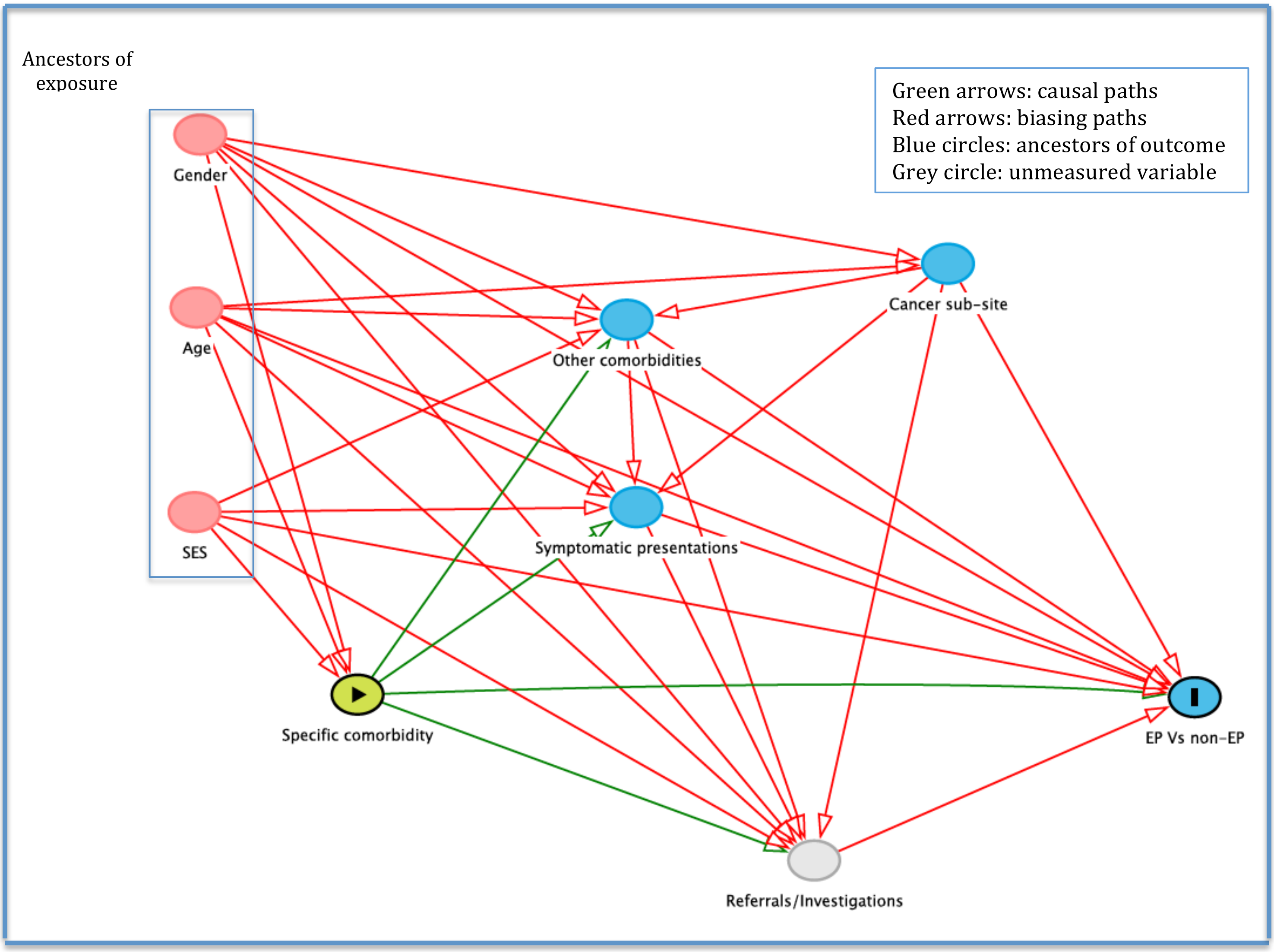

Causal paths (in green) linking comorbidities to emergency presentations can involve multiple mechanisms affecting:

Patients
- Symptom interpretation/attribution
- Help-seeking/reporting of symptoms
- Preferences for diagnostic tests
- Familiarity with healthcare system
- Opportunities to discuss symptoms
- Overall health status

Doctors
- Symptom interpretation/attribution
- Priorities

Healthcare system
- Guidelines
- Availability of services/tests
- Waiting list criteria for accessing services
- Duration of GP visits, booking systems, etc.

Potential-outcomes methods: evaluating the effect of comorbidities on the risk of colon cancer being diagnoses through emergency versus non-emergency routes

We estimated the average effect of comorbidities on emergency presentations in the population of colon cancer patients, which corresponds to the difference between the mean of the potential-outcomes (i.e. emergency presentations) we would expect if all individuals in the population were exposed (i.e. had the comorbidity) and the mean of the potential-outcomes if all individuals in the population were not exposed. Unbiased potential outcome means can be obtained under specific assumptions, including consistency of exposure, conditional exchangeability of treatment and positivity[12]. Similarly to recent cancer studies we used the doubly robust combinations of regression adjustment and inverse probability weighting (IPWRA)[11]. We modelled emergency presentation, our outcome, with socio-demographic characteristics and alarm symptoms as explanatory variables in a logit model. This model was combined with a second model predicting the exposure (comorbidity), using the same covariates as explanatory variables. Each comorbidity type ('serious' non-GI comorbidity, IBS/diverticular disease, gynaecological condition, hypertension) was considered as the exposure in successive models and in turn combined with the outcome model. Using the teffects Stata command, we estimated the Potential Outcome mean (POmean), corresponding to the risk of emergency presentation among non-comorbid cancer patients, as well as the 'average treatment effect' (ATE) of comorbidities on emergency presentations[10]. The risk of emergency presentation in the population of cancer patients with each specific comorbidity was calculated by summing the comorbidity-specific ATE and the corresponding POmean.
