# Supplementary material for: Contrasting effects of comorbidities on emergency colon cancer diagnosis: a longitudinal data-linkage study in England
Source: BMC Health Serv Res. 2019 May 15;19:311. doi: 10.1186/s12913-019-4075-4 (PMC6521448; doi:10.1186/s12913-019-4075-4)
Supplement: Supplementary file 2 — Prevalence of cancer alarm symptoms in the pre-diagnostic year among comorbid and non-comorbid patients diagnosed with colon cancer following emergency presentation (EP) (PDF 51 kb) [file 12913_2019_4075_MOESM2_ESM.pdf]

Table: Prevalence of cancer alarm symptoms in the pre-diagnostic year among comorbid and non-comorbid patients diagnosed with colon cancer following emergency presentation (EP)

	WOMEN			MEN		
	Cancer alarm symptoms	Patients with EP	p-value	Cancer alarm symptoms	Patients with EP	p-value
	%	Total=940		%	Total=874	
**Serious non-GI comorbidities recorded in secondary care°**						
0	20.2	618	0.899	10.6	530	<0.001
1+	19.9	322		20.6	344	
**Gynaecological conditions recorded in primary care^**						
Never	20.2	916	0.670			
New onset/chronic/past	16.7	24				
**Anxiety/depression recorded in primary care**						
Never	17.9	694	0.002	12.6	643	0.021
New onset pre-diagnostic year	35.9	53		22.1	68	
Chronic/past	23.8	193		19.0	163	
**IBS or diverticular disease recorded in primary care**						
Never	18.6	827	0.008	14.4	832	0.884
New onset pre-diagnostic year	32.1	56		15.0	20	
Chronic/past	29.8	57		18.2	22	
**Haemorrhoids recorded in primary care**						
Never	19.9	912	0.290	14.0	835	0.119
New onset pre-diagnostic year	42.9	7		30.0	10	
Chronic/past	23.8	21		24.1	29	
**Other benign GI conditions recorded in primary care^**						
Never	19.8	913	0.442	14.3	859	0.354
New onset pre-diagnostic year	25.0	4		20.0	5	
Chronic/past	30.4	23		30.0	10	
**Hypertension monitoring during the pre-diagnostic year**						
No	3.3	510	<0.001	1.0	527	<0.001
Yes	40.0	430		35.2	347	

°Serious non-gastrointestinal (non-GI) comorbidities include all the comorbidities recorded in secondary care, excluding peptic ulcer, liver disease, inflammatory bowel disease and ulcerative colitis.

^Due to sparse data new onset/chronic/past gynaecological conditions have been grouped together. Other benign GI conditions include coeliac disease, gallbladder diseases and others.
